# Unsupervised Learning of Progress Coordinates during Weighted Ensemble Simulations: Application to NTL9 Protein Folding

**DOI:** 10.1021/acs.jctc.4c01136

**Published:** 2025-03-19

**Authors:** Jeremy M. G. Leung, Nicolas C. Frazee, Alexander Brace, Anthony T. Bogetti, Arvind Ramanathan, Lillian T. Chong

**Affiliations:** † Department of Chemistry, University of Pittsburgh, Pittsburgh, Pennsylvania 15260, United States; ‡ Data Science and Learning Division, Argonne National Laboratory, Lemont, Illinois 60439, United States; § Department of Computer Science, University of Chicago, Chicago, Illinois 60637, United States

## Abstract

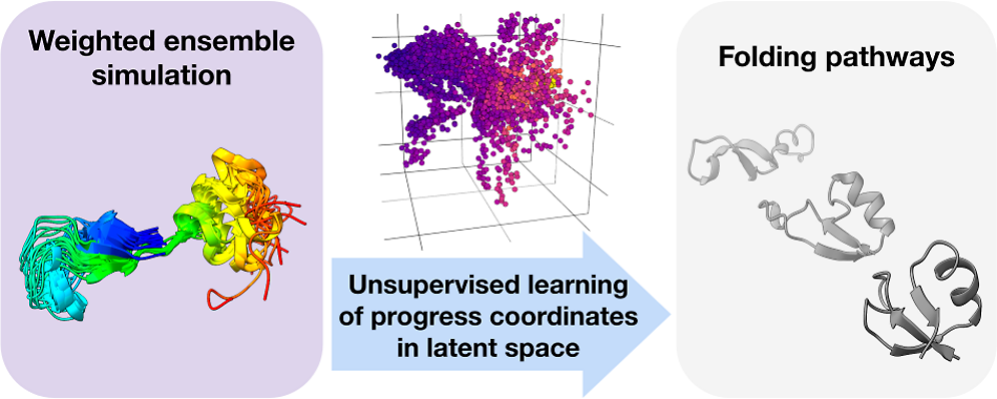

A major challenge for many rare-event sampling strategies is the identification of progress coordinates that capture the slowest relevant motions. Machine-learning methods that can identify progress coordinates in an unsupervised manner have therefore been of great interest to the simulation community. Here, we developed a general method for identifying progress coordinates “on-the-fly” during weighted ensemble (WE) rare-event sampling via deep learning (DL) of outliers among sampled conformations. Our method identifies outliers in a latent space model of the system’s sampled conformations that is periodically trained using a convolutional variational autoencoder. As a proof of principle, we applied our DL-enhanced WE method to simulate the NTL9 protein folding process. To enable rapid tests, our simulations propagated discrete-state synthetic molecular dynamics trajectories using a generative, fine-grained Markov state model. Results revealed that our on-the-fly DL of outliers enhanced the efficiency of WE by >3-fold in estimating the folding rate constant. Our efforts are a significant step forward in the unsupervised learning of slow coordinates during rare event sampling.

## 1 Introduction

Rare-event sampling methods have been increasingly used to simulate long-time-scale biological processes at the atomic level.^1−3^ For many of these methods, a major challenge that remains is the identification of a progress coordinate (also known as a reaction coordinate or collective variables) that captures the relevant slow motions. Given that the intrinsic dimensionality of a molecular dynamics (MD) simulation with N atoms is 3N-6 (in Cartesian coordinates), even relatively small systems can be challenging to analyze by using approaches that focus on motions along only a few dimensions. Strategies for identifying progress coordinates include the use of fast, approximate trajectories,^4^ identification of coordinates that correlate with the committor (or commitment probability),^5,6^ and automated artificial intelligence (AI) techniques such as machine and deep learning (DL).^7−12^

AI techniques can identify progress coordinates by detecting distinct conformational states in an unsupervised manner based solely on the atomic coordinates of structures sampled by an MD simulation. This detection is commonly facilitated by projecting the high-dimensional data from MD simulations onto low-dimensional manifolds containing a compressed representation of data. As demonstrated by a recent study, DL techniques involving the application of a convolutional variational autoencoder (CVAE) can identify effective progress coordinates for simulating the folding of small proteins via analysis of extensive MD simulations and the use of such progress coordinates with adaptive sampling has accelerated the sampling of folding events by >100× relative to conventional MD (cMD) simulations.^7,13^

**Figure 1:**
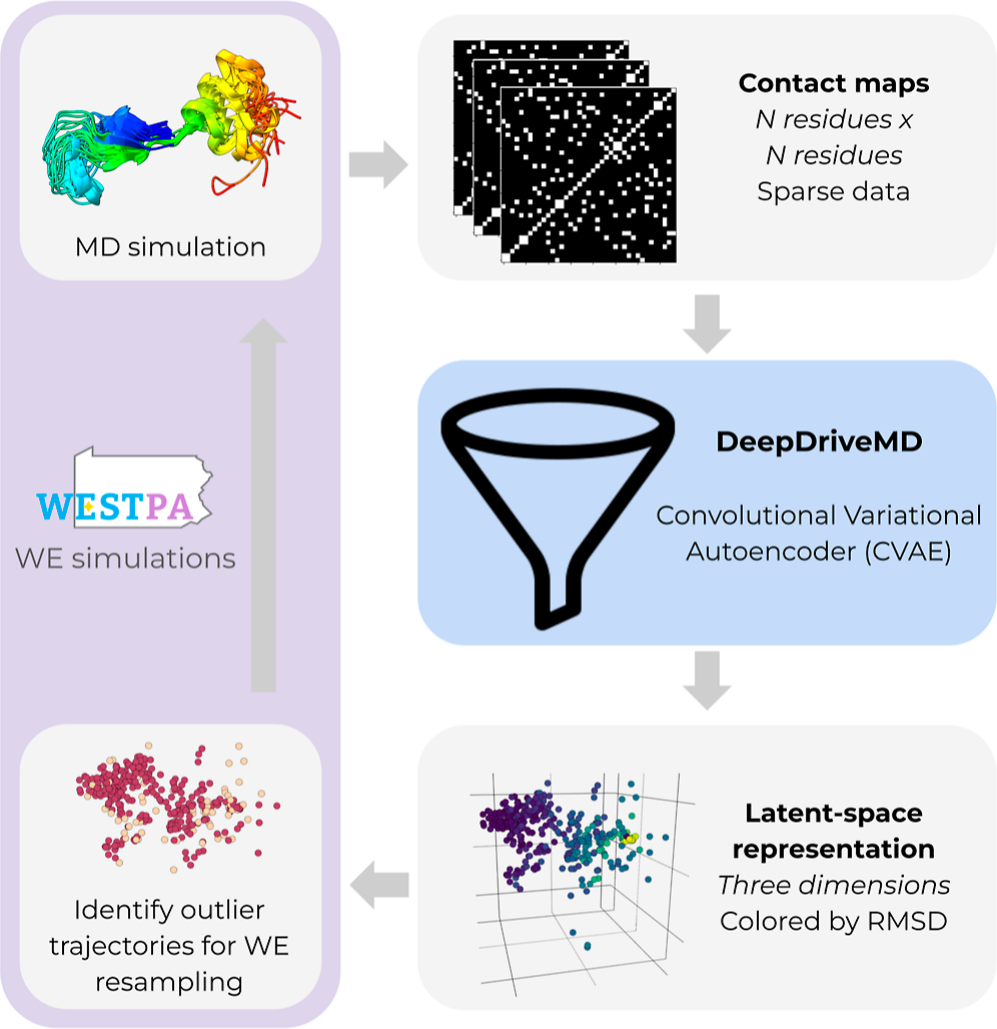
Workflow for DL-enhanced WE simulations. On-the-fly DL of progress coordinates during a WE simulation involves the application of a CVAE to compress the high-dimensional simulation data down to a three-dimensional latent space model. The high-dimensional data are in the form of pairwise residue contact matrices for selected conformations from the WE simulation. A WE resampling procedure is periodically applied by replicating outlier trajectories to enrich for sampling of rare, barrier-crossing transitions (e.g., protein folding). WE simulations are run using the WESTPA software,^18,19^ and the CVAE model^23^ is created using the DeepDriveMD software.^14−16^

Here, we have developed a DL method to learn progress coordinates “on-the-fly” during weighted ensemble (WE)^14−16^ rare-event sampling.^17−19^ WE is a path sampling strategy that has enabled atomistic simulations of complex processes such as protein folding,^20^ protein–ligand (un)binding,^21^ and large-scale conformational transitions in proteins.^22^ The DL method involves the application of a CVAE to compress high-dimensional WE simulation data down to lower-dimensional representations in latent space and then replicating outlier trajectories during a resampling procedure (Figure 1). CVAE models are particularly effective in anomaly detection through capturing spatial relationships between the pixels of an image.^23^

As a proof of principle, we applied our DL-enhanced WE strategy to simulate the folding process of the N-terminal domain of the L9 (NTL9) protein. Our simulations employed discrete-state synthetic molecular dynamics (synMD) trajectories,^24^ which are ideal for methods testing due to their greatly reduced computational cost, atomistic structures, and analytical “ground-truth” solution for steady-state observables (i.e., rate constants). We determine the features of the simulation data that are needed to build an effective latent space model of the system and train the latent space model “on-the-fly” to learn an effective progress coordinate for the molecular process of interest.

## 2 Methods

### 2.1 Overview of WE Path Sampling

WE path sampling enhances the efficiency of generating pathways and rates for rare events (e.g., protein folding and binding) by running a large number M of weighted trajectories in parallel and iteratively applying a resampling procedure at fixed time intervals τ.^17,25^ At each WE iteration, the resampling procedure involves replicating trajectories that have occupied less-visited regions of configurational space and occasionally terminating trajectories that have occupied more frequently visited regions. Such regions are typically defined as bins or clusters along a progress coordinate. For binned WE simulations, the goal is to provide equal sampling of each bin such that only trajectories within each bin are eligible to be merged together. For the binless WE simulation, as used for our DL-enhanced WE simulations, any trajectories may be merged together in order to maintain a fixed total number of trajectories for each WE iteration. Trajectory weights are tracked rigorously such that the weights sum to a total probability of one, thereby ensuring that no bias is introduced into the dynamics. To maintain a nonequilibrium steady state, trajectories that reach the target state (e.g., folded state for protein folding) are “recycled”, initiating a new trajectory from the initial state (e.g., unfolded state) with the same statistical weight.

### 2.2 DL-Enhanced WE Simulations of Protein Folding

To further enhance the efficiency of WE simulations in sampling rare events, we have developed a method that employs DL to learn progress coordinates on-the-fly during a WE simulation. All WE simulations were run using the WESTPA 2.0 software (https://github.com/westpa/westpa),^18^ in conjunction with synMD trajectories^24^ DL analysis was carried out using the mdlearn Python library associated with the DeepDriveMD software (https://github.com/ramanathanlab/mdlearn).^14−16,26^ The mdlearn library includes linear, nonlinear, and hybrid machine learning tools for learning latent space representations (embedding models) of MD simulation data to characterize biologically relevant conformational transitions.^27−31^ While the DeepDriveMD software orchestrates adaptive sampling using various MD engines, the mdlearn library provides support for ML/AI methods within the DeepDriveMD software.^15,16^

In our workflow for DL-enhanced WE simulations (Figure 1), the DeepDriveMD software compressed high-dimensional pairwise residue contact maps down to three-dimensional, latent space representations using a CVAE.^23^ In the contact maps, a pair of residues was considered to be in contact if the minimum distance between their C_α_ atoms was within 8 Å. While one might consider using continuous residue–residue distance matrices as input, we opted for binary contact maps, which are robust to minor structural variations, making them effective for studying conformational states and training models like variational autoencoders (VAEs).^32,33^

The DL-enhanced WE resampling procedure aims to replicate trajectories from selected “outlier” conformations. These conformations were identified using (i) the local outlier factor (LOF) anomaly detection method^34^ applied to CVAE latent space representations of trajectory data and (ii) a single structural feature of the protein system in real space, the C_α_ RMSD from the folded structure. The LOF method,^34^ implemented in scikit-learn,^35^ is an unsupervised learning algorithm that quantifies the extent to which a data point (conformation) deviates from its neighboring points based on variations in local density (LOF score; see Supporting Information).

**Figure 2:**
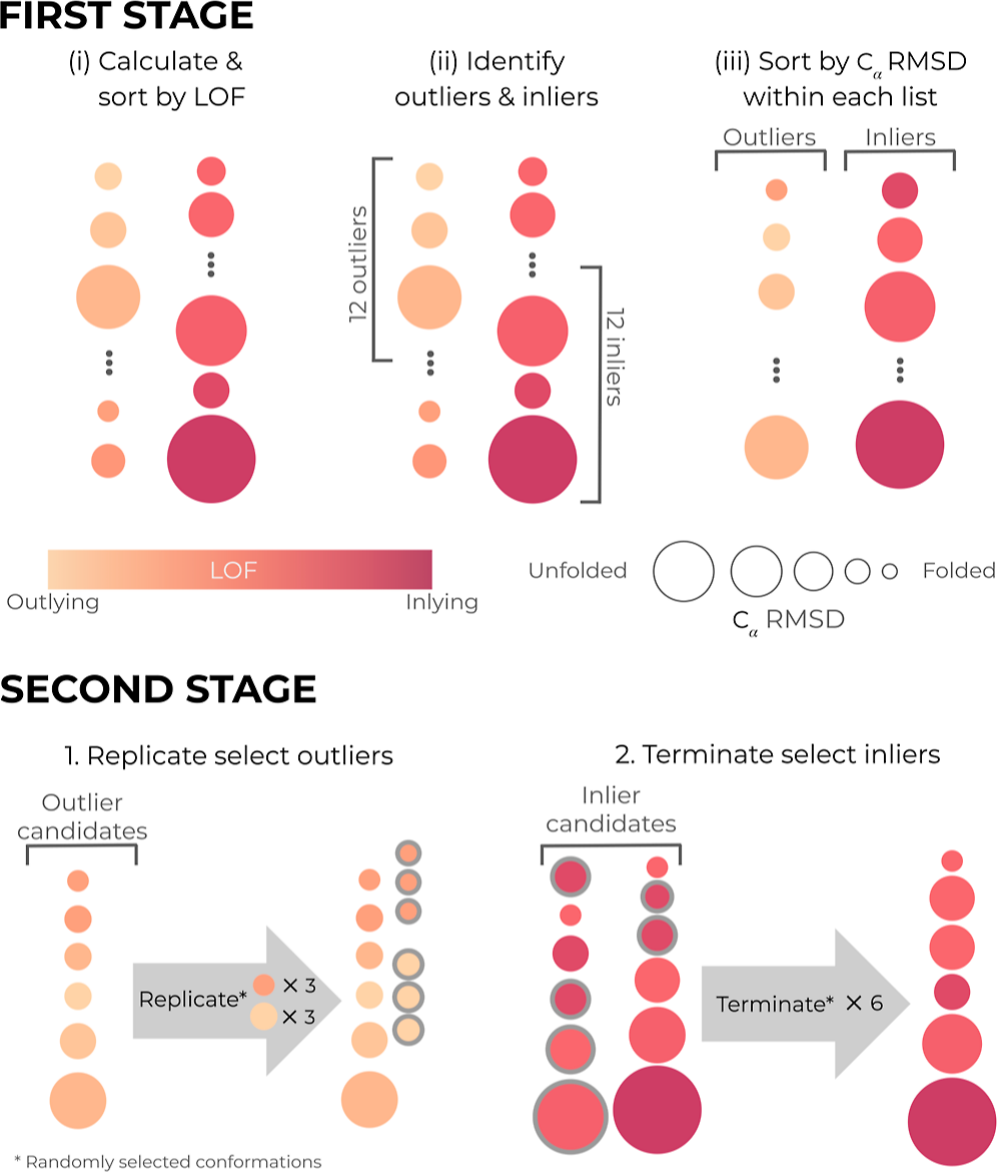
Illustration of the DL-enhanced WE resampling procedure. The WE resampling procedure was applied in two stages. In the first stage, trajectories were sorted by their LOF score, designating the top 12 trajectories as “outliers” and bottom 12 trajectories as “inliers”. In the second stage, a fixed number of *M* = 72 total trajectories was maintained through a random combination of replicating up to six lowest-RMSD trajectories and merging up to 12 highest-RMSD trajectories. The maximum numbers of replication and termination moves were each set to six (see Methods). Trajectory weights were rigorously tracked throughout the simulation.

The DL-enhanced WE resampling procedure was applied in two stages (Figure 2). In the first stage, we identified outliers among the *M* total trajectories at the current WE iteration by (i) sorting the trajectories by the LOF score, (ii) designating the top 12 trajectories as “outliers” (high LOF scores) and the bottom 12 as “inliers”, and (iii) ranking each list of trajectories by the C_α_ RMSD from the folded structure. To avoid generating trajectories with extremely low weights, trajectories with statistical weights beyond a minimum threshold of 1 × 10^–40^ were removed from the list of outliers. Likewise, to avoid a single trajectory with a majority of the total probability, a trajectory with a statistical weight beyond a maximum threshold of 0.1 was removed from the list of inliers. In the second stage, we applied the WE resampling procedure, replicating and terminating trajectories to maintain a fixed total number of *M* = 72 trajectories. Candidates for replication were the six outliers with the lowest C_α_ RMSD values and candidates for termination were the 12 inliers with the largest C_α_ RMSD values. For the outliers, there could be multiple ways to achieve six splits (e.g., split one trajectory into six, split two trajectories into three each, etc.). In the same way, there are also multiple ways to achieve six terminations (e.g., merge six trajectories into six other trajectories pairwise, merge six trajectories into one trajectory etc.). All possible ways to achieve six splits or terminations were considered, and the chosen method was randomly selected. For merges, in accordance with the rules of the WE protocol, the surviving trajectory in each termination group was randomly chosen based on trajectory weights. In this study, the maximum numbers of replication and termination instances were each set to six, but all parameters described above, including those for calculating the LOF score, can be customized by the users. Future studies will be conducted to further optimize the choice of parameters.

### 2.3 Propagation of synMD Trajectories

To enable rapid testing of each WE protocol with an atomistic system, we used the synMD approach^36^ to propagate discrete-state trajectories in a WE simulation. This approach involves propagating discrete-state Markov chain trajectories with a fixed time step among the “microbins” of a generative, fine-grained Markov state model (MSM) based on bin-to-bin transition probabilities. Here, our MSM was based on a set of cMD simulations of the NTL9 protein folding process (2.5 μs of total simulation time) with a lag time of 10 ps. These simulations employed the Amber ff14SB force field^37^ with generalized Born implicit solvent (Hawkins, Cramer, Truhlar model;^38,39^ igb = 1)^40^ and were performed in the *NVT* ensemble at 300 K using a weak Langevin thermostat (collision frequency of 5 ps^–1^).

The MSM was previously constructed by Russo and Zuckerman^41^ by first generating pairwise heavy-atom distance matrices of the simulation data, excluding nearest neighbors, and then applying the variational approach for Markov processes (VAMP)^42^ to reduce the dimensions of these matrices into 356 components covering 85% of the variance. Microbins of the MSM were generated by applying a stratified k-means clustering^36^ of the simulation data in which trajectories were clustered within “strata” bins defined along their C_α_ RMSD to a reference folded structure. Any microbins that did not involve any direct or indirect microbin-to-microbin transitions to the unfolded or folded states were removed, and their corresponding structures were reassigned to nearby surviving microbins. The resulting MSM consisted of 3512 microbins computed using a 10 ps lag time, which was reduced from 13,250 initial clusters (250 clusters per stratum). Stratified bin boundaries were positioned at 0.1 Å increments along [1.1, 4.5], 0.2 Å increments along [4.6, 6.4], and 0.3 Å increments along [6.6, 9.6]. The unfolded state was defined as having ≥9.6 Å C_α_ RMSD from a reference folded crystal structure (PDB 2HBB).^43^ The folded state was defined as having <1 Å C_α_ RMSD from the same reference structure.

For our WE simulations of NTL9 protein folding, synMD trajectories were propagated among the 3512 microbins of the MSM mentioned above using a resampling time interval τ of 10 ps. At each τ, the microbin that was visited by a trajectory was backmapped to a representative structure of that microbin (k-means cluster) to generate discrete trajectories of the NTL9 folding process. To maintain nonequilibrium steady state conditions, a trajectory reaching the target folded state was “recycled” by initiating a new trajectory from a randomly selected conformation of the initial unfolded-state ensemble with the same statistical weight. The initial unfolded-state ensemble consisted of 22 representative conformations, and the folded state consisted of a single structure. To generate the unfolded-state ensemble, we applied stratified k-means clustering as described above to the 2.5 μs cMD simulations of the NTL9 protein folding process to yield 22 clusters, and for each of these clusters, we selected the conformation closest to the center of the cluster. The resulting ensemble of initial unfolded conformations were assigned equal statistical weights.

### 2.4 Training of Convolutional Variational Autoencoder Models

The VAE is a deep neural network architecture that can be used for unsupervised learning of a continuous latent-variable model that captures salient features of a data set. A VAE consists of an encoder *q*_ϕ_(***z***|***x***^(*i*)^) that compresses input data ***x***^(*i*)^ into a small latent code ***z*** and a decoder *p*_θ_(***x***^**(i)**^|***z***) that reconstructs the code to its original form.^44^ VAEs are trained on a joint optimization objective function that attempts to minimize the reconstruction error of the input data and maximize the correspondence to a selected prior distribution *p*_θ_(***z***) (e.g., Gaussian) by computing the Kullback–Leibler (KL) divergence, which acts as a regularizer, via the loss function
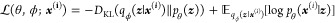
1

In this work, we employed a CVAE.^23^ The encoder network was parameterized with a series of four convolution layers each with 16 filters and a kernel size of 3 connected to a 128 dimensional linear layer with 0.5 dropout probability. The linear layer processed the flattened output tensor of the final convolutional layer, which together, reduced the 40 × 40 input contact matrix into a three-dimensional information bottleneck forming a latent space representation of the trajectory data following an approach similar to Romero et al.^45^ The decoder module mirrors the encoder using a series of transposed convolutions to parameterize the network. A rectified linear-unit (ReLU) activation function was used between each interior layer, transforming the final layer output via a sigmoid activation function to act as a Bernoulli distribution of the contact probability for each residue pair. As the contact map elements are binary, we computed the reconstruction loss by taking the binary cross-entropy loss of the predicted sigmoidal outputs against the ground truth contact. To regularize the model, we used a standard normal Gaussian distribution *N*(0, 1) prior for which a closed-form KL divergence was derived.^44^ The model was trained using the RMSprop optimization algorithm^46,47^ with a learning rate of 0.001 and minibatch size of 64 for 100 epochs (cycles of DL training) until convergence of the loss function and variance-bias trade-off (Figure S1). During inference, latent space conformer representations are directly computed as the encoded mean vector instead of the resampled vector used during training to ensure consistent and reproducible representations. CVAE models were implemented using the mdlearn Python library.^16^ Full details of the training data sets are as follows.

#### 2.4.1 Pretrained DL WE Simulations

For these simulations, a deep CVAE model was pretrained on representative conformations of each MSM microbin for NTL9 protein folding with the addition of 21 folded-state conformations. These conformations were generated using 21 1 ns of cMD simulations propagated from the single folded structure of our MSM. Given that the MSM only included a single folded conformation, the addition of 21 folded conformations was necessary to provide an equal number of conformations for the folded and unfolded states in the training set for the CVAE model (i.e., 22 conformations for each state).

#### 2.4.2 On-the-Fly DL WE Simulations

For these simulations, an initial CVAE model was trained on a base data set of 2000 conformations from 20 ns (2000 steps) of synMD trajectories combined with the 22 folded conformations mentioned above. A new CVAE model was then trained every 10 WE iterations by updating the base data set with data (contact maps) from the latter 50 WE iterations. This periodic updating of the training data set enabled the CVAE model to “learn” an improved internal latent space representation of the system as new regions of conformational space were explored.

#### 2.4.3 Use of CVAE and Alternative Models

CVAE offers a level of convenience that enables the learning of a simple, low-dimensional manifold that captures the intrinsic folding dimensions of the simulations explored here. As demonstrated in several applications,^23,28,48^ the CVAE-learned manifold can cluster the conformations from ensemble simulations in the latent space corresponding to biophysically relevant features. We also note several alternative approaches for the choice of the machine learning methods exist, including linear methods such as anharmonic conformational analysis^49^ or hybrid variants,^29^ and these methods could also be incorporated into the framework. We also note that other methods such as state predictive information bottleneck^50^ can be integrated into the framework. Moreover, previous work from our group has demonstrated that the CVAE-learned latent manifold provides robust information for subsequent stages of enhanced sampling workflows, including bounding the space for outlier detection,^15^ which can be challenging for DL methods.

### 2.5 Binless Control Simulations with Sorting by RMSD

To assess the impact of DL on the efficiency of our WE simulations, we performed bin-less control WE simulations without using the DL-based CVAE model to identify outlier trajectories. In these control simulations, trajectories were randomly shuffled before applying the WE resampling procedure, which was based on the C_α_ RMSD from the folded structure. The top six outlier trajectories were selected as candidates for splitting, and the bottom 12 inlier trajectories were candidates for merging. We also evaluated the effectiveness of sorting the trajectories solely by their LOF scores in CVAE latent space representations, without any additional ranking based on C_α_ RMSD from the folded structure.

### 2.6 Binned Control Simulations with an RMSD Progress Coordinate

As another point of comparison, we ran binned control WE simulations without the use of DL, employing a one-dimensional progress coordinate consisting of the C_α_ RMSD from the folded structure and rectilinear bins positioned using the minimal adaptive binning (MAB) scheme.^51^ We applied the MAB scheme with 10 rectilinear bins between the trailing and leading trajectories, up to 2 bins for the bottleneck and leading trajectories, and 6 target trajectories per bin to yield a similar total number of trajectories as the other WE protocols used in this study (*M* = 72 trajectories).

### 2.7 Calculation of the Folding Rate Constant

The folding rate constant *k*_fold_ was directly calculated from our WE simulations using the following exact Hill relation^52^
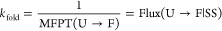
2where MFPT(U → F) is the mean first-passage time (average time) it takes for the protein to transition from the unfolded to the folded state and Flux(U → F|SS) is the nonequilibrium steady-state probability flux carried by trajectories originating from the unfolded state and reaching the target folded state. Uncertainties represent 95% credibility regions over 10 trials of WE simulation, as determined using a Bayesian bootstrap method.^53,54^ The ground-truth *k*_fold_ value was determined from our generative MSM model using the Deeptime Python library.^55^ The *k*_fold_ estimates in this study are based on simulations of NTL9 folding in implicit solvent with low solvent viscosity (collision frequency γ = 5 ps^–1^).^20^ Thus, while NTL9 folding occurs on the millisecond timescale at water-like viscosity (γ = 80 ps^–1^), it occurs on the microsecond timescale in our simulations.

### 2.8 Estimating DL-Enhancement of WE Efficiency

The efficiency *S*_k_ of a DL-enhanced WE simulation over a control WE simulation in computing a rate constant of interest (here, the folding rate constant *k*_fold_) was estimated using the following equation^17,56^
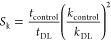
3where *t*_control/test_ is the total simulation time for a control/test simulation, respectively, and *k*_control/test_ is the relative error in the *k*_fold_ estimate (ratio of the width of the uncertainty of the rate constant relative to the value of the rate constant, where the uncertainty represents the 95% credibility region) for the corresponding simulations. Thus, the efficiency of a WE simulation in calculating the rate constant is determined by taking the ratio of the total simulation times for the control and test WE protocols that would be required to estimate the rate constant with the same relative error, assuming that the square of the width of the 95% credibility region on the rate constant is inversely proportional to the total simulation time.^56^

## 3 Results and Discussion

We have developed a WE simulation method that applies DL to learn an effective progress coordinate “on-the-fly” during a simulation. The DL process involves identifying outlier trajectories based on a LOF anomaly score in latent space and the C_α_ RMSD from the target state in real space. Our benchmark application is the simulation of the NTL9 protein folding process using discrete-state synMD trajectories. To assess the impact of DL on the efficiency of the WE simulations, we ran control WE simulations without DL using (i) a “binless” approach where trajectories are sorted by the C_α_ RMSD from the folded state and (ii) a rectilinear, adaptive binning approach along a one-dimensional progress coordinate consisting of the C_α_ RMSD from the reference folded structure. We also determined the effectiveness of applying DL on-the-fly during a WE simulation vs pretraining on cMD simulation data prior to running a WE simulation. Key details of all WE simulation protocols used in this study are summarized in Table 1.

**Table 1:** WE Simulation Protocols Used in This Study^a^

WE protocol	outlier identification	deep-learning (DL) training
on-the-fly DL	by LOF and RMSD	every 10 WE iterations on data from the latter 50 WE iterations
pretrained DL	by LOF and RMSD	once from 2.5 μs cMD simulations
binless control	by RMSD	none
binned control	by RMSD	none

a For each WE protocol, we summarize the criteria for identifying outlier trajectories and simulation data used for DL training. WE simulations using either pre-trained or on-the-fly DL identified outlier trajectories in a “binless” manner based on the LOF score in a three-dimensional CVAE latent space model of the system and C_α_ RMSD from the folded structure in real space. Two types of control simulations were run without the use of DL: (i) binless control simulations where outlier trajectories were identified based on the C_α_ RMSD from the folded structure, and (ii) binned control simulations where adaptive binning was applied along a progress coordinate consisting of the C_α_ RMSD from the folded structure.

### 3.1 Unsupervised Learning Identifies Unfolded, Intermediate, and Folded States

**Figure 3:**
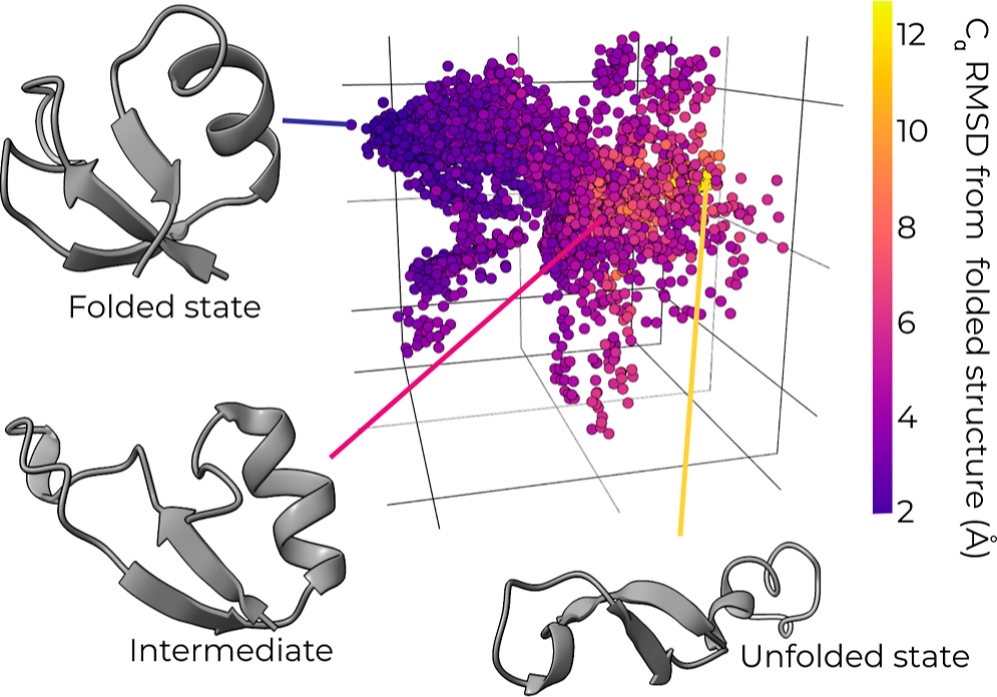
Pretrained CVAE model identifies key states for the NTL9 protein folding process. A three-dimensional CVAE latent space model pretrained using a NTL9-folding simulation data set with data points colored by the C_α_ RMSD from the folded structure. The training data set was generated using a set of representative structures for the microbins of a MSM (one structure for each microbin) that was constructed using 2.5 μs total simulation time of cMD simulations with conformations saved every 10 ps and an additional 21 folded state structures generated from 21 ns of cMD simulations from a folded state structure. This pretrained CVAE model separates key states of the NTL9 folding process, revealing unfolded, intermediate, and folded states.

Before applying on-the-fly DL during a WE simulation, we verified that a CVAE latent space representation of data from a set of cMD simulations of the NTL9 folding process (2.5 μs of total simulation time) could identify key stable or metastable states. As shown in Figure 3, a three-dimensional CVAE representation of the simulation data was sufficient for this identification when the data were colored according to the C_α_ RMSD from the folded structure.

### 3.2 Real-Space Structural Metric Is Necessary to Identify Outliers

**Figure 4:**
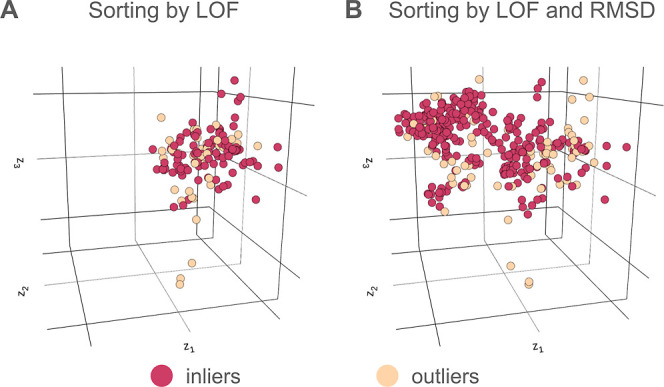
A real-space structural metric is necessary to identify productive outliers in latent space. Three-dimensional CVAE latent space representations of the NTL9 folding process based on pretrained DL with (A) sorting by only the LOF score and (B) sorting by both LOF score and a real-space metric (C_α_ RMSD from the folded structure). Conformations identified as outliers are colored yellow and those identified as inliers are colored red.

**Figure 5:**
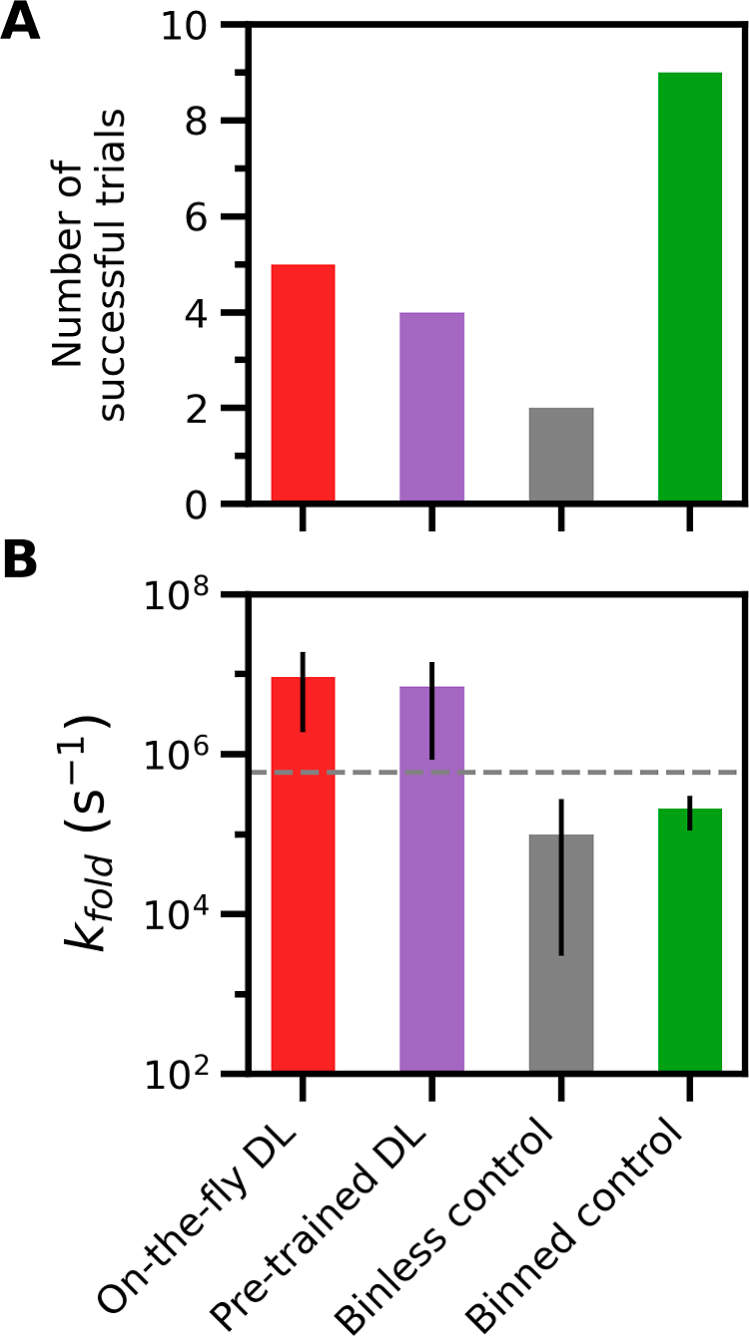
Number of successful simulation trials and average *k*_fold_ estimates generated by each WE protocol. (A) Number of successful trials for each WE protocol. A trial was considered successful if the *k*_fold_ estimate was within 1 order of magnitude of the ground-truth value [dashed horizontal line in (B)]. (B) The average *k*_fold_ estimate generated by each WE protocol. Uncertainties represent 95% credibility regions over 10 trials for each WE protocol, as determined using a Bayesian bootstrap method.^53,54^ Data shown for each WE protocol are based on the same total simulation time of 14.5 μs.

Our results revealed that the sorting of trajectories by the LOF score in latent space was not sufficient for efficient generation of successful folding events and that additional sorting using a real-space structural metric (i.e., RMSD) was necessary. When only sorting by the LOF score, the WE simulations sampled primarily the unfolded state (high-RMSD region; Figure 4A). On the other hand, additional sorting by RMSD resulted in extensive sampling of latent space and the identification of outlier conformations along the periphery (Figure 4B). This additional sorting more than doubles the number of successful folding events by replicating trajectories at the leading edge while terminating trajectories at the trailing edge (Figures 5A and S2). Furthermore, binless control simulations (without DL) with sorting of trajectories by only RMSD were able to generate successful events, while those with random sorting of trajectories were unable to generate any successful events (Figures S3–S4).

### 3.3 On-the-Fly DL Enhances WE Efficiency

We next tested the effectiveness of the on-the-fly DL of a progress coordinate during a WE simulation of the NTL9 folding process. Compared to the binless control simulations, pretrained DL simulations were 3-fold more efficient in estimating a *k*_fold_ value (Table 2). On-the-fly DL simulations were also more efficient, but to a smaller extent (2.2-fold), partially because our estimate of the efficiency includes the 2.5 μs in aggregate of cMD simulations used for DL training. Both on-the-fly and pretrained DL simulations exhibited a substantially lower variance in the rate-estimates relative to the binless control simulations with the same total simulation time (Figures 5B and S5).

**Table 2:** Efficiency of DL-Enhanced versus Control WE Simulations^a^

WE protocol	*k*_fold_ (s^–1^)	Δ*k*	total simulation time (μs)	*S*_k_ relative to binless control	*S*_k_ relative to binned control
on-the-fly DL	3.0 × 10^5^	2.7	8.3	2.2	1.3
pretrained DL	6.7 × 10^5^	2.5	7.2	3.0	1.8
binless control	6.3 × 10^4^	2.1	30.9	1.0	0.6
binned control	4.5 × 10^5^	1.3	46.3	1.7	1.0

a The efficiency *S*_k_ is estimated by taking the ratio of total simulation times for the DL-enhanced vs. binless or binned control WE simulations that would be required to estimate the rate constant *k*_fold_ with the same relative error Δ_k_, which is the ratio of the width of the 95% credibility region on *k*_fold_ and the estimated value of *k*_fold_ (see Methods).^17,56^ The total simulation time for pre-trained DL simulations includes time invested for the cMD simulations used for DL training. All simulations were run until the ground-truth value fell within their corresponding 95% credibility regions (Figure S7).

We also compared the efficiency of our DL-enhanced WE simulations relative to binned control WE simulations employing the MAB scheme (see Methods),^51^ which has been shown to efficiently surmount large barriers. We applied this adaptive binning scheme along a one-dimensional progress coordinate consisting of the C_α_ RMSD from a reference folded structure. The use of DL also showed a marginal increase in efficiency compared to the binned control simulations, with a 1.3-fold gain for on-the-fly DL and 1.8-fold gain for pretrained DL (Table 2). Among all the WE protocols, the adaptively binned WE simulations were the most efficient in generating initial folding events (Figure S2) but did not reach a steady state that yields the ground-truth *k*_fold_ value. The DL-enhanced WE simulations were reasonably converged, reaching the ground-truth value within the same total simulation time.

Compared to the binned control simulations, the greater efficiency of both the on-the-fly and pretrained DL-enhanced WE simulations in reaching the ground truth appears to be due to their “binless” nature. These binless strategies allow us to allocate a majority of the M trajectories for exploitation toward the target state, potentially leading to faster convergence to a steady state that yields the ground-truth *k*_fold_ value. However, these strategies resulted in a relatively wide range of trajectory weights, yielding abrupt “ramp-up” times in the kinetics in contrast to the exponential ramp-up times that are characteristic of a binned strategy (Figures S5–S6)^57^ and thereby relatively large variances in the folding rate estimates between trials (Figure 5A). On the other hand, the WE simulations with adaptive binning resulted in a more narrow range of trajectory weights (Figure S8) with slower convergence to a steady state but lower variance in rate estimates between trials.

### 3.4 Overhead of DL Training

We note that the reported efficiencies (*S*_k_ values) for our DL-enhanced WE simulations do not include the overhead for training the CVAE model. With the exception of the pretrained DL protocol, a single trial of each WE protocol was completed within minutes to hours, highlighting the advantage of using synMD trajectories for rapid testing in methods development. Although the wall-clock time for a pretrained DL simulation was only 0.22 h for running the WE of synMD trajectories, ∼30 h was required to complete the cMD simulations for pretrained the CVAE model (Table 3). On the other hand, the on-the-fly DL simulations required only a small initial data set (here, 20 ns of synMD trajectories). Relative to the binless and binned control simulations, the >20-fold longer wall-clock time of the on-the-fly DL simulations is due to the substantial overhead for training the deep CVAE models. For future simulation studies, we recommend starting with on-the-fly DL WE simulations to generate initial successful pathways for a rare-event process of interest, then running additional WE trials using the final updated DL model. We note that the training data used for our pretrained model does not accurately represent the steady-state distribution. As a result, neither the pretrained nor on-the-fly DL protocol accurately captures the upper bound for simulation performance. The simulation performance can be further optimized by optimizing various WE and LOF parameters as described in the Methods.

**Table 3:** Wall-Clock Times for Each WE Protocol^a^

WE protocol	wall-clock time (hrs)
on-the-fly DL	1.57
pretrained DL	30.22
binless control	0.08
binned control	0.05

a Wall-clock times required for running a single WE trial simulation with 1.45 μs total simulation time and any DL training. Each simulation was run using a single thread of an AMD Ryzen 9 7950X CPU. DL training was performed on a single NVIDIA RTX 4090 GPU.

### 3.5 Comparisons of Binless and Binned Strategies

As is evident in our results, binless strategies have certain strengths and limitations relative to binned strategies. Binned strategies provide even coverage of the state space by maintaining a target number of trajectories per bin. However, such strategies require a rapidly increasing total number of trajectories as the simulation progresses. On the other hand, binless strategies maintain a fixed total number of trajectories but result in uneven coverage of state space. In terms of maintaining trajectory information, binned strategies merge only trajectories within the same bin, while binless strategies might merge trajectories that occupy distant regions of state space and reduce the number of distinct trajectories. While binless strategies are more efficient in generating continuous pathways, due to uneven coverage and loss of distinct trajectories, binless strategies may overestimate rates with larger variation between WE trials while binned strategies can provide convergence to accurate rates depending on the timescale of the process (Figure S7).^58^ To improve binless strategies for more accurate and precise rate estimates, one can increase the number of total trajectories, as well as modify the criteria for merging trajectories to prevent any loss of distinct trajectory information.

## 4 Conclusions

We have developed a WE path sampling method that applies DL on-the-fly to learn effective progress coordinates during a simulation. Our DL-enhanced WE method learns progress coordinates by identifying outlier trajectories based on relatively low local densities in latent space, as quantified by LOF scores and structural information in real space (RMSD from the target structure). We applied our method to simulations of the NTL9 protein folding process using discrete-state synMD trajectories.

Our “binless” WE method was ∼3-fold more efficient than binless control simulations with no DL and 1.8-fold more efficient than binned control simulations with no DL. These gains in efficiency underscore the value of projecting high-dimensional simulation data onto a low-dimensional latent space model for identifying progress coordinates that are effective for rare-event sampling. It is worth noting that our reported efficiency gains account for only the total simulation times and not for the overhead of training the DL models. To reduce this overhead, we have been integrating the WESTPA software with the Colmena framework^59,60^ to implement model-training in parallel with the execution of WE simulations (unpublished work).

While our binned control simulations achieve the highest precision in rate-constant estimates, these simulations do not reach the ground-truth rate constant within the same total simulation time as that used for our on-the-fly DL protocol. On the other hand, the on-the-fly DL protocol reaches the ground truth, but with a higher variance in the rate-constant estimates. The necessity of using a real-space RMSD metric in addition to the latent space LOF score highlights the challenge of identifying productive outlier conformations in latent space without a physically intuitive structural metric. Finally, we note that the DL method used here represents a simple prototype, and future versions of our framework will allow the integration of techniques such as information bottleneck,^50^ Deep-TICA,^61^ and other techniques.^10^”.

## Data Availability

All input files and scripts needed to run and analyze the WE simulations in this study are provided in the GitHub repository: https://github.com/westpa/DL-enhancedWE and deposited on Zenodo under DOI: 10.5281/zenodo.13387514.

## Supporting Information Available

Additional details on calculation of the local outlier factor score; training loss vs. training epoch for the pre-trained CVAE model; time-evolution plots of minimum C_α_ RMSD from the folding structure, number of successful trials, and *k*_fold_ estimates for each WE simulation protocol; histogram of trajectory weights for each WE protocol (PDF)
