# Supplementary material for: Unsupervised Learning of Progress Coordinates during Weighted Ensemble Simulations: Application to NTL9 Protein Folding
Source: J Chem Theory Comput. 2025 Mar 19;21(7):3691–9. doi: 10.1021/acs.jctc.4c01136 (PMC11983707; doi:10.1021/acs.jctc.4c01136)
Supplement: Supplementary file 1 — ct4c01136_si_001.pdf [file ct4c01136_si_001.pdf]

Supporting Information: Unsupervised Learning of Progress Coordinates During Weighted Ensemble Simulations: Application to NTL9 Protein Folding

Jeremy M. G. Leung,[†,§] Nicolas C. Frazee,[†,§] Alexander Brace,[‡,¶,§] Anthony T. Bogetti,[†] Arvind Ramanathan,[*,‡,¶] and Lillian T. Chong[*,†]

†*Department of Chemistry, University of Pittsburgh, Pittsburgh, Pennsylvania 15260, United States*

‡*Data Science and Learning Division, Argonne National Laboratory, Lemont, Illinois 60439, United States*

¶*Department of Computer Science, University of Chicago, Chicago, Illinois 60637, United States*

§*Contributed equally to this work*

E-mail: ltchong@pitt.edu; ramanathana@anl.gov

1 Supplementary Information

Calculation of the Local Outlier Factor score. The Local Outlier Factor (LOF) score of a given conformation p was defined as:[S1]

$$LOF\ SCORE(p) = \frac{\sum_{o \epsilon N(p)} \frac{LRD(o)}{LRD(p)}}{|N(p)|} \tag{1}$$

where $N(p)$ is the set of k nearest neighbors of point p, while $LRD(p)$ and $LRD(o)$ are the local reachability densities (LRDs) of p and neighboring points $o\ \epsilon N(p)$, respectively. An LOF score close to 1 indicates that point p is not an outlier. While the LRD is defined as the inverse of the average reachability distance of p to its k-nearest neighbors, the maximum distance is used instead to prevent close neighbors from disproportionately influencing the average.[S1,S2] In the present study, the distance between any two conformations A and B, with corresponding latent space coordinates $\bar{A}$ and $\bar{B}$, was calculated using a cosine distance, given by $1 - cos(\theta) = 1 - \frac{\bar{A} \cdot \bar{B}}{|\bar{A}||\bar{B}|}$.[S3] A cosine distance was chosen over Euclidean distance because, in a nonlinear latent space, vector directionality is more meaningful than magnitude. The set $N(p)$ included at least $k = 20$ nearest neighbors in the latent space, with up to 1,000 randomly selected conformations from all previous weighted ensemble (WE) iterations. If $N(p)$ contained more than 20 neighbors, the additional points corresponded to unique conformations at the same maximum distance from p.

List of Figures

S1 Training loss as a function of training epoch for the pre-trained CVAE model S-4

S2 Time-evolution of the minimum C_α RMSD from the folding structure reached by each WE protocol S-5

S3 Number of successful trials for each WE protocol S-6

S4 Average folding-rate constant kfold estimates for each WE protocol S-7

S5 Time-evolution of the average folding-rate constant k_{fold} estimate for each WE simulation protocol. S-7

S6 Time-evolution of the folding-rate constant k_{fold} estimate for each of the 10 simulations run for each WE protocol S-8

S7 Time-evolution of the average folding-rate constant k_{fold} estimate S-9

S8 Histogram of trajectory weights for each WE protocol S-10

2 Supplementary Figures

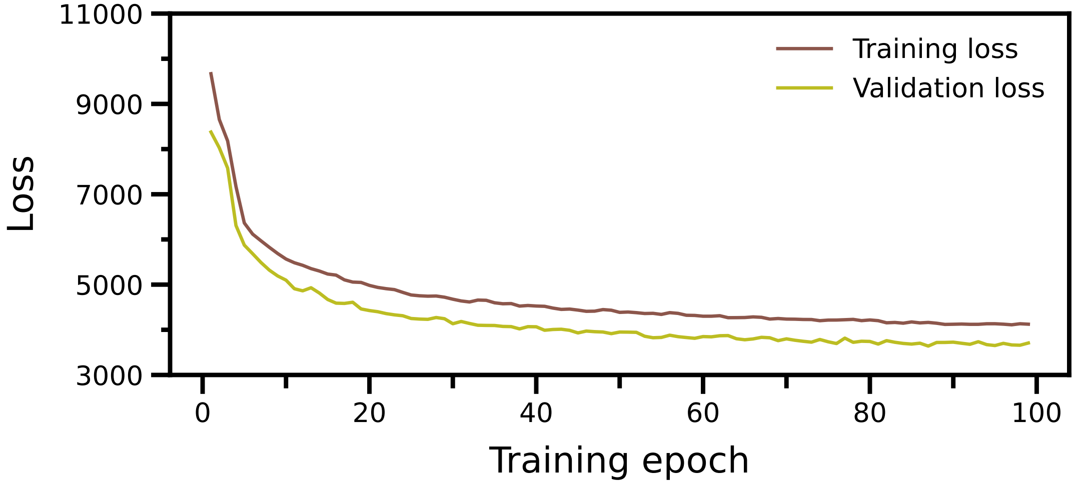

Figure S1: **Training loss as a function of training epoch for the pre-trained CVAE model.** Training and validation losses for the CVAE model converged after 100 training epochs (cycles of DL training). The training loss appears higher than the validation loss because the latter was measured at the end of an epoch whereas the former was averaged over each training step of the epoch.

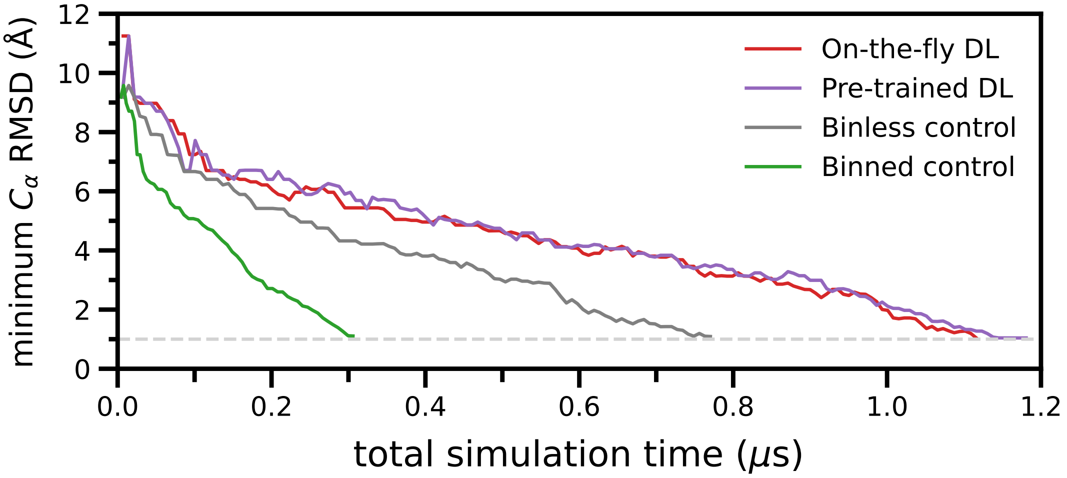

Figure S2: **Time-evolution of the minimum C_α RMSD from the folding structure reached by each WE protocol.** The data trace for each WE protocol was truncated at the total simulation time where a folding event was first generated (dotted line).

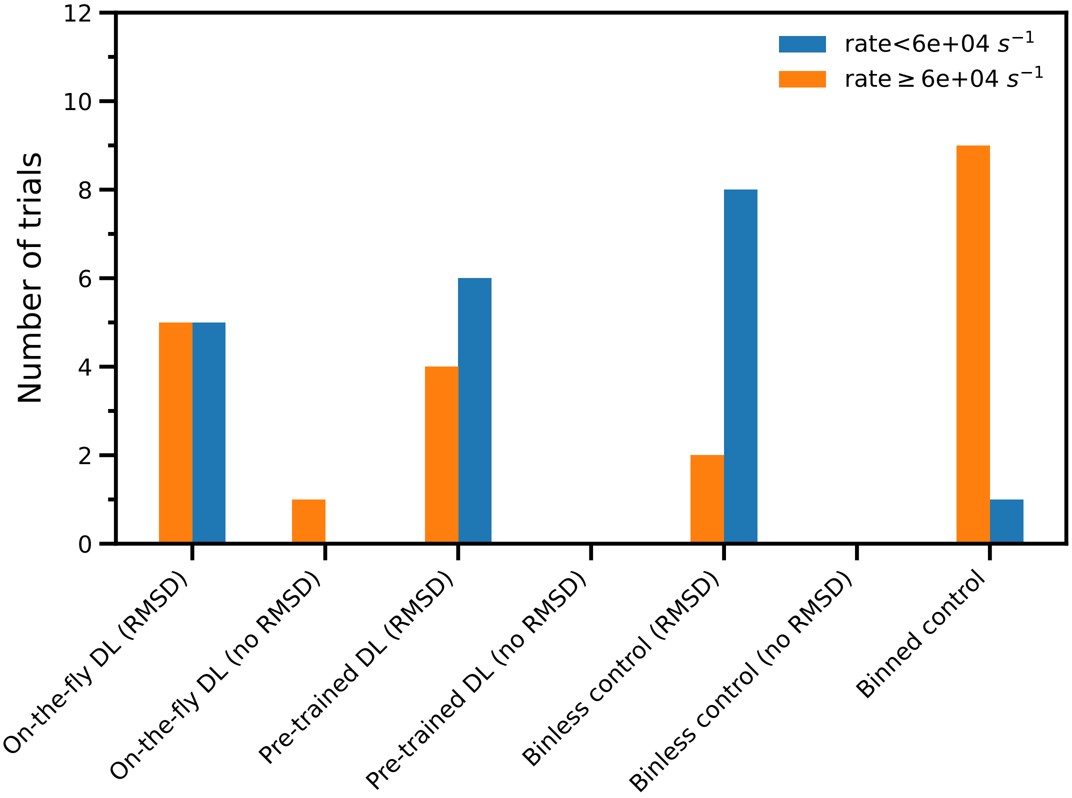

Figure S3: **Number of successful trials for each WE protocol.** Orange bars indicate trials with folding-rate constant k_{fold} estimates within one order of magnitude of the ground-truth value, whereas blue bars indicate trials that have not yet reached the ground-truth value (beyond one order of magnitude). The use of a real-space structural metric (i.e., C_α RMSD from the folded structure) to further sort trajectories prior to WE resampling yielded a greater number of trials with successful events.

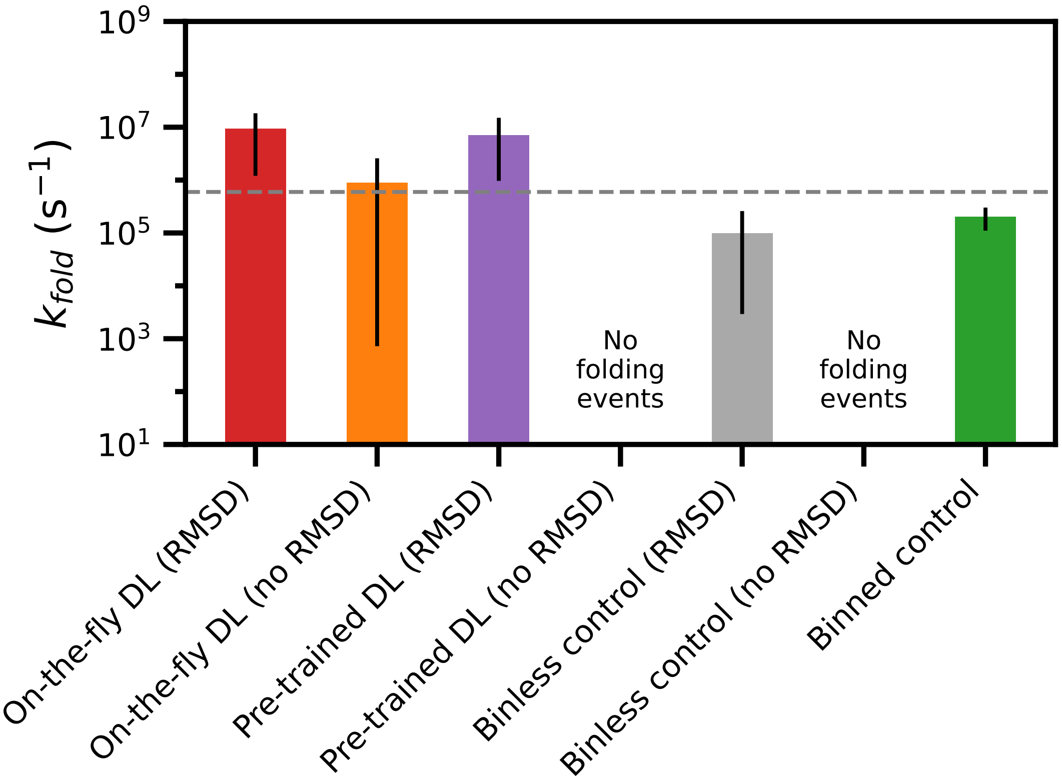

Figure S4: **Average folding-rate constant k_{fold} estimates for each WE protocol.** Uncertainties represent 95% credibility regions over 10 trials with each WE protocol, as determined using a Bayesian bootstrap method.[S4,S5] The ground-truth value is shown as the dashed horizontal line. The total simulation time for each simulation protocol was 14.5 µs.

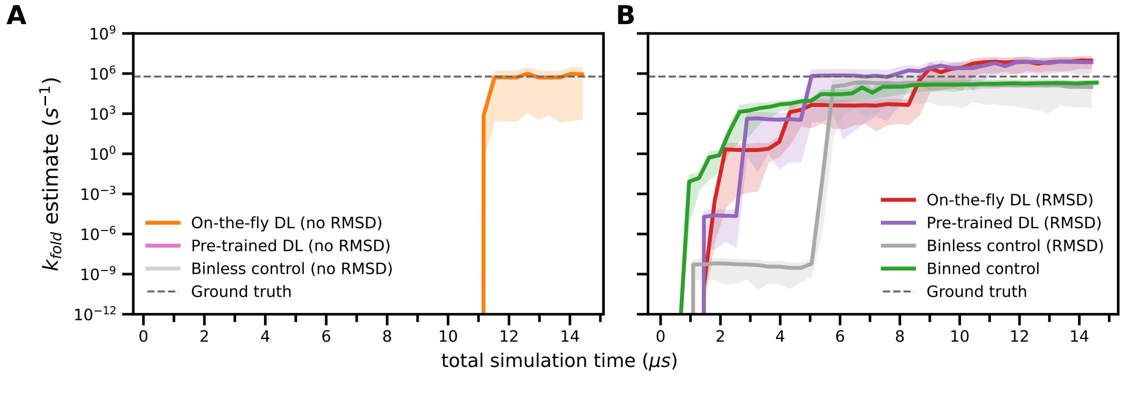

Figure S5: **Time-evolution of the average folding-rate constant k_{fold} estimate for each WE simulation protocol.** (A) Folding-rate constant k_{fold} estimate of three protocols with random sorting of trajectories (instead of sorting by real-space RMSD) prior to WE resampling. Binless control simulations (without RMSD sorting) were unable to generate any successful folding events within a total simulation time of 14.5 µs. (B) Folding-rate constant k_{fold} estimates using four WE protocols with sorting of trajectories by real-space RMSD prior to WE resampling.

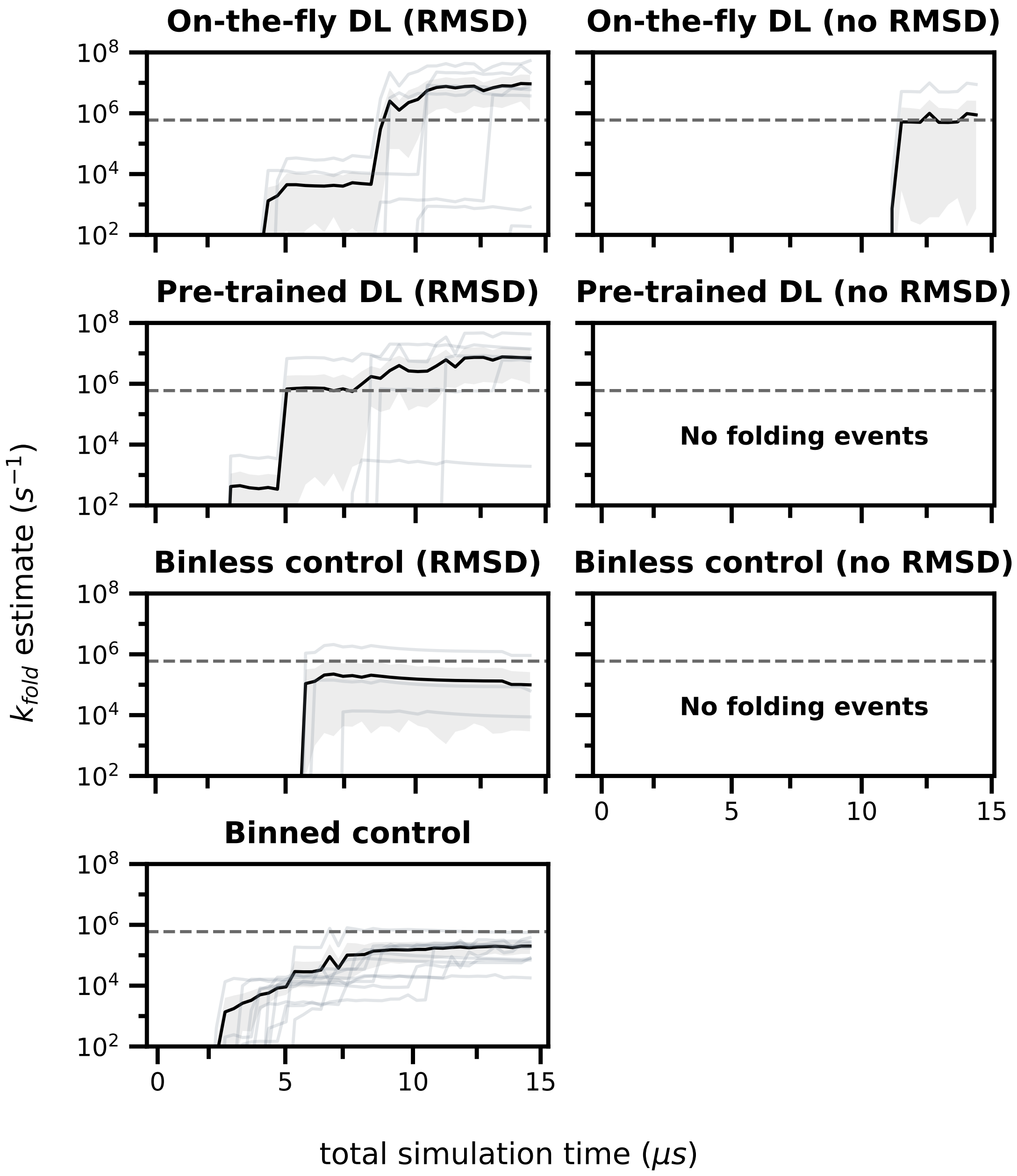

Figure S6: **Time-evolution of the folding-rate constant k_{fold} estimate for each of the 10 simulations run for each WE protocol.** Each panel represents the folding-rate constant estimation for a single WE protocol. The gray traces represent individual trials and the black trace represents the average of the 10 trials with a 95% credibility region (gray shading), where the x-axis should be interpreted in terms of units of 100 ns. The horizontal dashed line represents the ground-truth value.

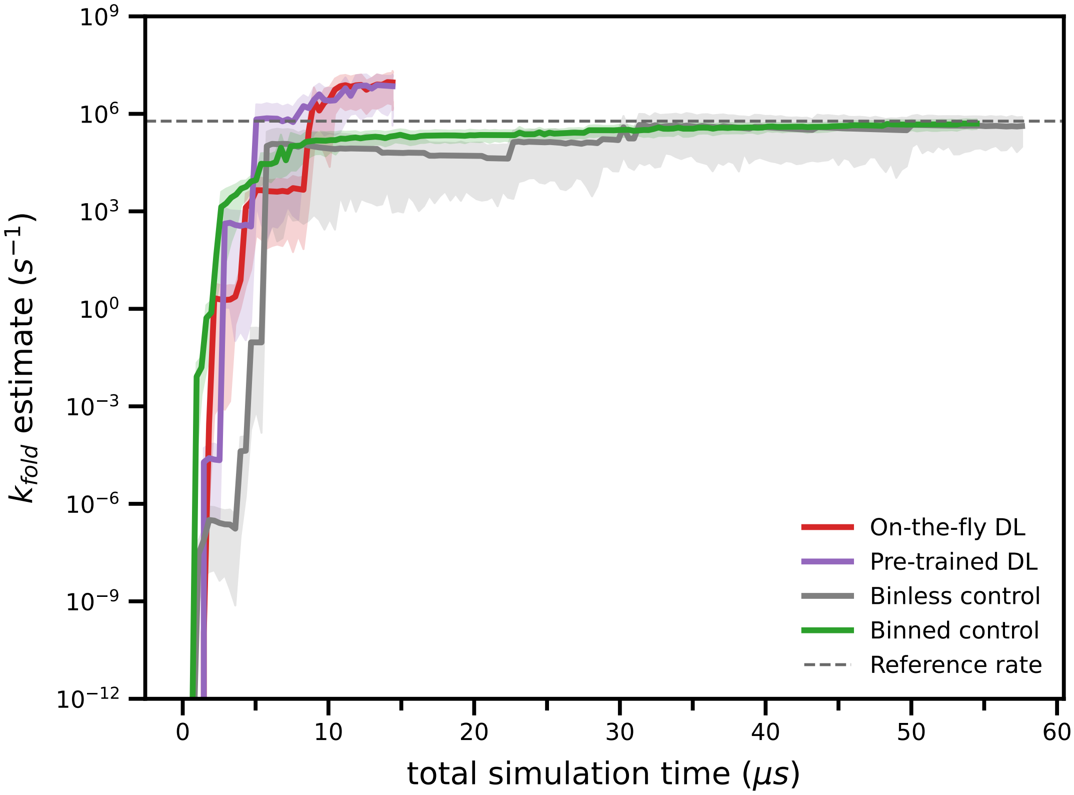

Figure S7: **Time-evolution of the average folding rate-constant k_{fold} estimate.** All simulations were extended until reaching the ground-truth value for the folding-rate constant k_{fold}. Compared to the DL methods, binned control simulations required 5-12x the amount of total simulation time and reached the ground-truth value with a lower uncertainty to reach the ground-truth value (95% credibility region).

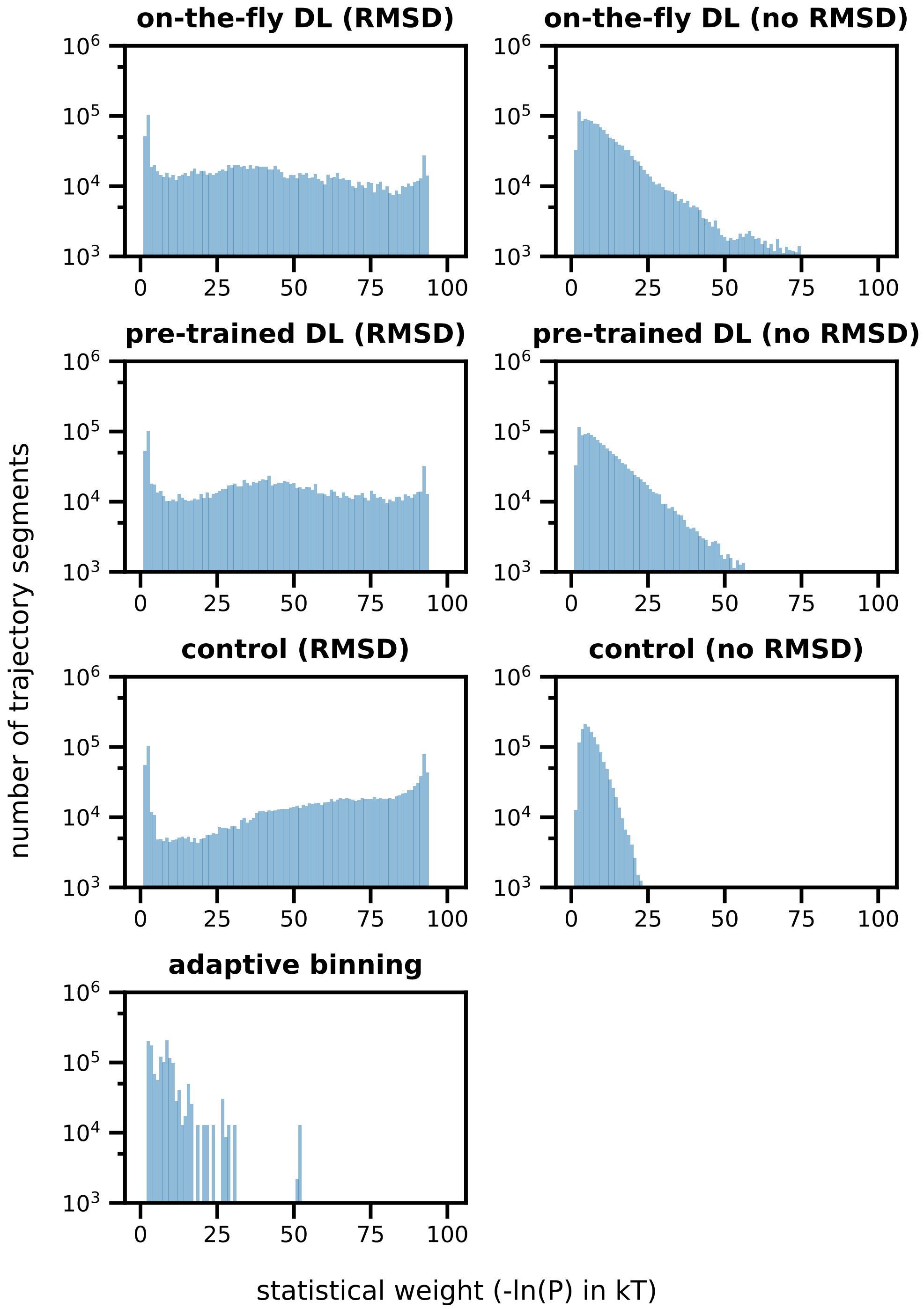

Figure S8: **Histogram of trajectory weights for each WE protocol.** With the exception of the binless control (no RMSD) simulations, the "binless" resampling protocols (top three rows) generated a wide range of trajectory weights. In contrast, the binned control simulations generated a narrower range of trajectory weights.

References

(S1) Breunig, M. M.; Kriegel, H.-P.; Ng, R. T.; Sander, J. LOF: identifying density-based local outliers. Proceedings of the 2000 ACM SIGMOD international conference on Management of data. New York, NY, USA, 2000; pp 93–104.

(S2) Pedregosa, F.; Varoquaux, G.; Gramfort, A.; Michel, V.; Thirion, B.; Grisel, O.; Blondel, M.; Prettenhofer, P.; Weiss, R.; Dubourg, V.; Vanderplas, J.; Passos, A.; Cournapeau, D. Scikit-learn: Machine Learning in Python. *MACHINE LEARNING IN PYTHON* **2007**,

(S3) Douze, M.; Guzhva, A.; Deng, C.; Johnson, J.; Szilvasy, G.; Mazaré, P.-E.; Lomeli, M.; Hosseini, L.; Jégou, H. The Faiss library. **2024**,

(S4) Mostofian, B.; Zuckerman, D. M. Statistical Uncertainty Analysis for Small-Sample, High Log-Variance Data: Cautions for Bootstrapping and Bayesian Bootstrapping. *Journal of Chemical Theory and Computation* **2019**, *15*, 3499–3509.

(S5) Rubin, D. B. The Bayesian Bootstrap. *The Annals of Statistics* **1981**, *9*, 130–134.
